# A progressive three-phase innovation to medical education in the United States

**DOI:** 10.1080/10872981.2018.1427988

**Published:** 2018-01-20

**Authors:** Cory M. Pfeifer

**Affiliations:** ^a^ Department of Radiology, University of Texas Southwestern Medical Center, Dallas, TX, USA

**Keywords:** Accelerated medical curriculum, educational innovation, undergraduate medical education

## Abstract

The practice of medicine has changed greatly over the past 100 years, yet the structure of undergraduate medical education has evolved very little. Many schools have modified their curricula to incorporate problem-based learning and organ systems-based curricula, but few schools have adequately addressed rising tuition costs. Undergraduate medical education has become cost-prohibitive for students interested in primary care. In the meanwhile, the concept of a separate dedicated intern year is outdated and mired in waste despite remaining a requirement for several hospital-based and surgical specialties. Described here is an innovative approach to medical education which reduces tuition costs and maximizes efficiency, based on principals already employed by several schools. This integrated curriculum, first suggested by the author in 2010, keeps the current USMLE system in place, exposes medical students to patient care earlier, expands and incorporates the ‘intern’ year into a four-year medical training program, provides more time for students to decide on a specialty, and allows residency programs to acquire fully-licensed practitioners with greater clinical experience than the status quo.

**Abbreviations:** MCAT: Medical college admission test; USMLE: US medical licensing examination

## Introduction

Over 100 years have passed since Abraham Flexner released the report that led to the framework by which most four-year medical schools still operate. Just as changes have been applied to both the Medical College Admission Test (MCAT) [1] and the US Medical Licensing Examination (USMLE) [2] to reflect modern trends in examination preparation and testing science, the need to consider widespread changes to the way we train physicians has never been more vital. It was not long ago that medical students simply studied notes obtained in a traditional classroom format, and the economical and lifestyle fates of medical students did not rely so heavily on the results of standardized testing. As technology has markedly increased the number of educational resources available in this test-anxiety-laden climate, many students feel as though a nationally standardized preclinical curriculum may now be preferable to their school’s more traditional curriculum [3].

In response to skyrocketing tuition and fees, the average medical student debt among indebted students was cited as $190,694 in 2017 [4]. According to the American Association of Medical Colleges (AAMC), tuition and fees made up only 3.8% of medical school revenue in 2016 on average [5]. The pursuit of medicine is becoming cost prohibitive and risk-laden. The surge of hospital-based practices reflects a change in the attitudes of modern doctors, and primary care and rural medicine have fallen out of favor, creating jobs for mid-level providers but resulting in an overall maldistribution of physicians.

The traditional medical curriculum in the USA (Table 1) requires two years of preclinical didactics (often separated by a dormant summer) followed by the first step of the USMLE, a year of required clinical clerkships (many of which involve a nationally-standardized ‘shelf’ exam), and a final year which is of variable content but is generally dominated by electives. In most cases, a sub-internship and two to three other required rotations are standard in the fourth year, while greater than 60% of the year is designed at the discretion of the student [6]. A survey of graduates of the 2011 Colorado School of Medicine showed that 57.1% of respondents rated the item ‘to take time off or have more time for myself’ as a very important purpose for the fourth year while an additional 29.3% agreed that this is a somewhat important purpose [7].

**Table 1.** Traditional curriculum. The first two years are centered on classroom didactics, and the first step of the USMLE is taken during the summer before clerkships begin. The core clerkships take place during the third year, and the second step of the USMLE is generally taken during the final year. The area designated in black is variable between medical schools and may contain downtime, offsite rotations, electives geared toward the actual practice of medicine in the first post-graduate year, and/or personal enrichment courses. The first post-graduate year can either be the first year of a residency program (as is the case in primary care specialties) or exist as a standalone preliminary year (as is common in many hospital-based and surgical specialties). Residency programs vary with respect to when the third step of the USMLE must be taken, but it is shown at the end of the first post-graduate year here to indicate that it is required – often in addition to at least one full year of post-graduate supervised practice – before a medical license may be issued.

The classical method of delivering the preclinical sequence has centered on study of the normal functions of the human body during the first year and the aspects of human disease the second year, but many schools have shifted toward organ systems-based blocks in which the anatomy, physiology, and pathology of an organ system is addressed before moving to then next system. Traditional lectures have in many instances been supplanted by more active learning methods [8]. The two parts of USMLE Step 2 are usually taken during the fourth year but are not required to apply for residency. The Electronic Residency Application Service (ERAS) opens for submissions in September of the fourth year, more than eight months prior to graduation.

While the length of residency training has not significantly changed in recent decades, the concept of having a dedicated intern year distinct from the rest of residency is falling out of favor. The persistence of a standalone post-graduate year 1 (PGY-1) no longer exists for primary care specialties and is nearly extinct for psychiatry. Neurology, anesthesiology, and physical medicine and rehabilitation are trending toward phasing them out in response to the desire for greater standardization and/or integration of this training [9]. The third step of the USMLE is most often taken during PGY-1, and passing is required for an unrestricted medical license. An unrestricted license also typically requires at least one year of post-graduate residency training for graduates of US medical schools [10]. As such, medical school graduates are functionally unemployable as doctors immediately following graduation. To meet the demand for rural doctors, Arkansas, Kansas, and Missouri have approached the concept of ‘assistant physicians,’ medical school graduates practicing medicine in collaboration with a fully-licensed practitioner [11].

## Modern adaptations to the traditional model

Nuanced curriculum adaptations have been in place for several years at competitive medical schools such as Baylor College of Medicine and Duke School of Medicine in which the preclinical sequence is delivered in less than two years. Others have evolved to meet student demand for more self-study by truncating the sequence to allow for a dedicated ‘capstone’ course or similar experience in the spring of the second year to permit self-preparation for USMLE 1. The newly opened Dell Medical School in Austin, Texas employs a 48-week preclinical curriculum [12]. The University of Texas Southwestern Medical Center now ends its preclinical coursework in the middle of the second year [13].

Some institutions have decreased the entire curriculum from four to three years on the basis of prior training or scholastic achievement/preparedness or in exchange for a commitment to enter a primary care residency [14]. The Family Medicine Accelerated Track (FMAT) at Texas Tech University not only removes the fourth year but provides scholarship funding equivalent to one year of tuition and fees [15]. Interestingly, Benson et al. found that primary care aspirants were significantly more likely to believe that the fourth year was an important means to broaden the educational experience when compared to those not going into primary care [16].

## The integrated curriculum: a three-phase four-year approach

Prior to the growing movement of three-year medical curricula that has taken place over the past seven years, a four-year, three-phase solution designed to address modern concerns – here referred to as the Pfeifer curriculum – was submitted to the academic medical community in 2011. While to date not attempted, medical schools that have implemented three-year curricula have validated the Pfeifer curriculum as a de facto proof-of-concept. This plan furthermore incorporates some of the elements later recommended by Ezekiel Emanuel and Victor Fuchs in 2012 [17].

The Pfeifer integrated curriculum (Table 2) reduces waste in preclinical education. The core phase decreases preclinical didactics from 24 months to 15 months by eliminating the summer break between the first and second years and by utilizing the value-oriented preclinical approaches in place at the Texas and North Carolina schools discussed above. Students would be engaged with patients earlier in training, a concept that physician associate programs have employed for years but has seemed contrary to the traditional Flexner medical sequence. While the example core components in this integrated curriculum reflect a systems-based approach to learning, the 15-month core phase could also be used for a subject-based system (e.g., anatomy, physiology, histology, etc.). The last three months are presented as a flexible integrative capstone experience leading up to USMLE 1 which is now common.

**Table 2.** Pfeifer integrated curriculum. The 15-month core (preclinical) phase is followed by a month break to take the first step of the USMLE. A 15-month clerkship phase ensues, essentially combining the traditional third year with the typical perfunctory required fourth year rotations. Another free month allows for both components of USMLE 2. The intern phase is now 16 months in duration. Of note, the example core and clerkship rotations are presented in alphabetical order.

The clerkship phase of the Pfeifer plan includes the currently required third and fourth year rotations and also allows for elective time. The final segment of the clerkship phase designated as ‘enrichment/electives’ corresponds to the variable rotations currently required in the fourth year and are customizable based on the demands of the individual school. Popular fourth-year requirements include critical care medicine, ambulatory medicine, and sub-internship. These could easily be included among the 15-month clerkship sequence. Having fewer months of paid tuition decreases the financial burden for those who are dissuaded from primary care careers by the amount of medical education debt.

The intent of keeping the new 16-month intern phase at the same institution as the medical school would allow for the student to have a ‘major’ (e.g., surgery, internal medicine, family medicine, etc.) which would designate which department provides the appropriately-accredited framework for the year and may result in inter-student competition for a desirable track but would also guarantee greater local retention for the initial graduate medical education experience. Students would have more clinical exposure to ascertain whether the career path they chose is the appropriate fit for them before a more permanent decision is made, a decision which fixes the time-period for which full reimbursement for training is allocated by the Centers for Medicare and Medicaid Services. In the Pfeifer curriculum, the individual would have completed six months of intern training before the application season opens in September of the final year.

The age of the separate PGY-1 year would dissolve along with the added expense of separate interviews and the current controversy associated with PGY-2-level surgical programs (e.g., urology) penalized for subtly requiring applicants to sidestep the match algorithm by agreeing to specific local PGY-1 preliminary year programs. Trainees in states requiring a year of supervised practice would be eligible for a full license by the end of the four-year sequence thus allowing the rebirth of the ‘general practitioner’ concept for interested individuals, as it exists in many other countries.

Since the Pfeifer curriculum is no longer centered on all trainees operating on a common academic year calendar, the ‘July effect’ whereby new medical student clerks start at the same time as newly minted PGY-1 interns would no longer apply. In fact, the new system builds in overlap of interns which may allow for improved subspecialty elective opportunities and increased clinical service to teaching hospitals.

For those who complete the Pfeifer curriculum and opt to pursue a full residency, programs would benefit from having enrollees with greater clinical experience as well as USMLE 1 and 2 scores on record for more uniform comparison of applicants. Likewise, applicants would have an improved understanding of their career choice and would be subject to closer professional relationships with attending physicians, resulting in more fruitful recommendation letters. Residency programs could require passage of USMLE 3 prior to matriculation which would alleviate the current risk of enrolling a trainee who is unable to succeed on the exam.

## Objections

The primary liability to the shortened undergraduate medical pathway cited by Cangiarella et al. was ‘reduced readiness for PGY-appropriate independence’ [18]. This assumes that students are actively engaged in learning activities that make them more independent with respect to clinical medicine during the current fourth year which seems counterintuitive to a year that includes so few requirements. Under the Pfeifer plan, the current truly clinical rotations in the clerkship phase remain in place. An additional cited potential liability is the note that ‘premature commitment to a specialty’ may come into place with a contracted curriculum. In the Pfeifer plan, trainees would have a broader clinical experience that begins sooner than most three-year plans, and applicants do not choose a residency until their ability to compete with fellow students has already been assessed. While concerns over maturity and personal development in shorter curricula have been raised, we must remember that other medical professionals are deemed mature enough to independently interact with patients at an earlier age than board-certified physicians. The current system includes much proverbial down time that can hardly be attributable to improved maturity with respect to patient interaction.

Even though medical schools would seemingly be the biggest losers under the Pfeifer curriculum with respect to unrealized medical tuition, two thirds of 154 surveyed deans endorsed debt reduction as a definite benefit of a shortened curriculum [18]. Many medical school faculty members, particularly those working in the community who donate their time solely to promote interest in ambulatory primary care, are usually not compensated for their teaching. The ‘away rotation’ concept common to fourth year students also results in donated teaching from cooperating schools. Likewise, many medical schools train students at branch campuses which receive even less tuition subsidy than their flagship counterparts and rely more heavily on community volunteers to provide elective training experiences. If a medical student’s fate is essentially decided eight months prior to graduation, and most of the fourth-year learning experiences are not required as part of a standard curriculum and/or offered via pro bono teaching, how are we not simply price gouging our students under the status quo? Admittedly, they are willing to pay for it as part of the time-honored lackadaisical ‘fourth-year spirit’ tradition, but supporting the practice is pushing our trainees into an unsuspecting world of debt. Mid-level provider training has ramped up in response to increased demands for healthcare services while medical schools have refused to abandon traditionally wasteful aspects of training.

Omitted from this discussion is the plight of the international medical graduate (IMG) who would have to find a way to integrate into this system. Mandating this 16-month internship program as a separate step for those entering from outside the USA, however, may improve standards for residency program entry by requiring this additional training experience. If the Pfeifer curriculum is implemented on a widespread basis, it seems more likely that community centers of graduate medical education would partner with nearby medical schools to provide an appropriately accredited intern experience to medical trainees.

Pundits of liberal medical education, expansion of public health content in the fourth year, and increased research activities have well-intentioned motivations, but what the USA needs right now are doctors who can provide clinical medical services. A very strong case can be made that physicians do not get enough training in the business of medicine, socioeconomic aspects of disease, or scientifically-oriented thinking, however, integrating these concepts longitudinally into graduate medical education would go further to benefit physician training than including these elements as requirements for trainees who are not even yet able to prescribe medicine. We simply can no longer justify these educational costs that are growing beyond control and discouraging students from choosing primary care.

## Conclusion

The Pfeifer curriculum offers a new way to build on the successes of accelerated preclinical pathways and capitalize on the benefits of modern three-year medical curriculum plans. Through the reduction of wasteful elements of the current system, a new paradigm in which all three steps of the USMLE can be taken within a four-year framework would emerge.

## References

[1] Mitchell K, Lewis RS, Satterfield J, et al The new medical college admission test: implications for teaching psychology. Am Psychol. 2016;71:125–5. 26866988 10.1037/a0039975 PMC5561549

[2] New features in step 1 and step 2 CK examinations. [cited 2017 111]. Available from: http://www.usmle.org/announcements/?ContentId=174

[3] London DA, Kwon R, Atluru A, et al More on how USMLE Step 1 scores are challenging academic medicine. Acad Med. 2016;5:609–610. 10.1097/ACM.0000000000001159 27115655

[4] Association of American Medical Colleges Medical student education: debt, costs, and loan repayment fact card. [cited 2017 111]. Available from: https://members.aamc.org/iweb/upload/2017%20Debt%20Fact%20Card.pdf

[5] Association of American Medical Colleges Revenues supporting programs and activities at fully-accredited U.S. medical schools FY2016. [cited 2017 111]. Available from: https://www.aamc.org/download/480304/data/fy2016_medical_school_financial_tables.pdf

[6] Barzansky B, Simon FA, Brotherton SE. The fourth-year medical curriculum: has anything changed in 20 years? Acad Med. 2001;76(Suppl 10):S36–8. 11597867 10.1097/00001888-200110001-00013

[7] Wolf SJ, Lockspeiser TM, Gong J Students’ perspectives on the fourth year of medical school: a mixed-methods analysis. Acad Med. 2014;89:602–607. 24556778 10.1097/ACM.0000000000000183 PMC4885563

[8] Brandi K, Schneid SD, Smith S, et al Small group activities within academic communities improve the connectedness of students and faculty. Med Teach. 2017;25:1–7. 10.1080/0142159X.2017.1317728 28440094

[9] Pfeifer CM Evolution of the preliminary clinical year and the case for a categorical diagnostic radiology residency. J Am Coll Radiol. 2016;13:842–848. 27162044 10.1016/j.jacr.2016.02.034

[10] Susser S Chart of physician licensing requirements by state. [cited 2017 111]. Available from: http://www.visalaw.com/wp-content/uploads/2014/10/physicianchart.pdf

[11] Bailey M Looming question for medical students: will they be shut out of advanced training? [cited 2017 111]. Available from: https://www.statnews.com/2016/03/17/medical-students-match-day/[cited

[12] Leading EDGE four-year curriculum. [cited 2017 11 1]. Available from: https://dellmed.utexas.edu/curriculum

[13] Foundation for Excellence Curriculum [cited 2017 November 1]. Available from: http://www.utsouthwestern.edu/education/medical-school/academics/curriculum/

[14] Raymond JR, Kerschner JE, Hueston WJ, et al The merits and challenges of three-year medical school curricula: time for an evidence-based discussion. Acad Med. 2015;90:1318–1323. 26266464 10.1097/ACM.0000000000000862 PMC4585483

[15] Family medicine accelerated track fact sheet. [cited 2017 111]. Available from: https://www.ttuhsc.edu/som/fammed/fmat/documents/FMAT_FactSheetJan2015.pdf

[16] Benson NM, Stickle TR, Raszka WV Going “fourth” from medical school: fourth-year medical students’ perspectives on the fourth year of medical school. Acad Med. 2015;90:1386–1393. 27002891 10.1097/ACM.0000000000000802

[17] Emanuel EJ, Fuchs VR Shortening medical training by 30%. JAMA. 2012;307:1143–1144. 22436952 10.1001/jama.2012.292

[18] Cangiarella J, Gillespie C, Shea JA, et al Accelerating medical education: a survey of deans and program directors. Med Educ Online. 2016;21:31794. 10.3402/meo.v21.31794 PMC4908065 27301381

